# A Positive Fluid Balance in the First Week Was Associated With Increased Long-Term Mortality in Critically Ill Patients: A Retrospective Cohort Study

**DOI:** 10.3389/fmed.2022.727103

**Published:** 2022-03-03

**Authors:** Tsai-Jung Wang, Kai-Chih Pai, Chun-Te Huang, Li-Ting Wong, Minn-Shyan Wang, Chun-Ming Lai, Cheng-Hsu Chen, Chieh-Liang Wu, Wen-Cheng Chao

**Affiliations:** ^1^Department of Critical Care Medicine, Taichung Veterans General Hospital, Taichung, Taiwan; ^2^Division of Nephrology, Department of Internal Medicine, Taichung Veterans General Hospital, Taichung, Taiwan; ^3^College of Engineering, Tunghai University, Taichung, Taiwan; ^4^Cloud Innovation School, Tunghai University, Taichung, Taiwan; ^5^Department of Medical Research, Taichung Veterans General Hospital, Taichung, Taiwan; ^6^Artificial Intelligence Workshop, Taichung Veterans General Hospital, Taichung, Taiwan; ^7^Department of Computer Science, Tunghai University, Taichung, Taiwan; ^8^Department of Automatic Control Engineering, Feng Chia University, Taichung, Taiwan; ^9^Big Data Center, Chung Hsing University, Taichung, Taiwan

**Keywords:** acute kidney injury, critical care, cumulative fluid balance, fluid balance, long-term outcome, mortality, shock

## Abstract

**Introduction:**

Early fluid balance has been found to affect short-term mortality in critically ill patients; however, there is little knowledge regarding the association between early cumulative fluid balance (CFB) and long-term mortality. This study aims to determine the distinct association between CFB day 1–3 (CFB 1–3) and day 4–7 (CFB 4–7) and long-term mortality in critically ill patients.

**Patients and Methods:**

This study was conducted at Taichung Veterans General Hospital, a tertiary care referral center in central Taiwan, by linking the hospital critical care data warehouse 2015–2019 and death registry data of the Taiwanese National Health Research Database. The patients followed up until deceased or the end of the study on 31 December 2019. We use the log-rank test to examine the association between CFB 1–3 and CFB 4–7 with long-term mortality and multivariable Cox regression to identify independent predictors during index admission for long-term mortality in critically ill patients.

**Results:**

A total of 4,610 patients were evaluated. The mean age was 66.4 ± 16.4 years, where 63.8% were men. In patients without shock, a positive CFB 4–7, but not CFB 1–3, was associated with 1-year mortality, while a positive CFB 1–3 and CFB 4–7 had a consistent and excess hazard of 1-year mortality among critically ill patients with shock. The multivariate Cox proportional hazard regression model identified that CFB 1–3 and CFB 4–7 (with per 1-liter increment, HR: 1.047 and 1.094; 95% CI 1.037–1.058 and 1.080–1.108, respectively) were independently associated with high long-term mortality in critically ill patients after adjustment of relevant covariates, including disease severity and the presence of shock.

**Conclusions:**

We found that the fluid balance in the first week, especially on days 4–7, appears to be an early predictor for long-term mortality in critically ill patients. More studies are needed to validate our findings and elucidate underlying mechanisms.

## Introduction

Fluid homeostasis is frequently altered in critical illness, and fluid balance has been attributed as one of the fundamental managements in patients admitted to the intensive care unit (ICU) (1). Fluid resuscitation is necessary to maintain tissue perfusion and improve cardiac output (2); however, excessive fluid accumulation may lead to detrimental effects, including prolonged tissue edema, impaired oxygen transport, reduced metabolite diffusion, and damaged cell–cell interactions (3). Increasing evidence has suggested that a positive fluid balance in the early stage of ICU admission might deteriorate outcomes in critically ill patients (4–7). One recent meta-analysis found that a positive cumulative fluid balance (CFB) in the first 3 days of ICU stay was associated with high hospital mortality [Relative Risk 2.15 (95% CI, 1.51–3.07)] (8). However, the optimal management of post-resuscitation fluid management, such as the day 4–7 fluid balance, remains unclear. Moreover, few studies have explored the association between early fluid balance and long-term survival (9).

Despite steady improvements in short-term mortality among critically ill patients, post-discharge mortality remains high (10). Among the ICU survivors, 15%−21% may die within the following year (11, 12), with ~6–8% mortality per year in the subsequent 5 years (12). Therefore, long-term outcomes in critical medical illnesses, have become more important as more patients survive acute illness. Thus, the research priorities of critical care medicine have expanded to not only save lives while patients are in the ICU but also toward a goal of understanding and improving long-term outcomes. Nevertheless, outcome ascertainment of CFB in the meta-analysis was somehow restricted to short-term outcomes consisting of ICU mortality, hospital mortality, and 3-month mortality (8); data regarding the long-term effects of fluid balance on survival in medical ICU was sparse.

We linked data from the National Health Insurance Research Database (NHIRD) in Taiwan and Electronic Medical Records at Taichung Veterans General Hospital (TCVGH) to establish a critical care database. This database enabled us to test the hypothesis that a higher CFB at day 1–3 and day 4–7 might be associated with an increased risk of long-term mortality in patients admitted to the ICU.

## Materials and Methods

### Study Population and Ethics Approval

**Figure 1:**
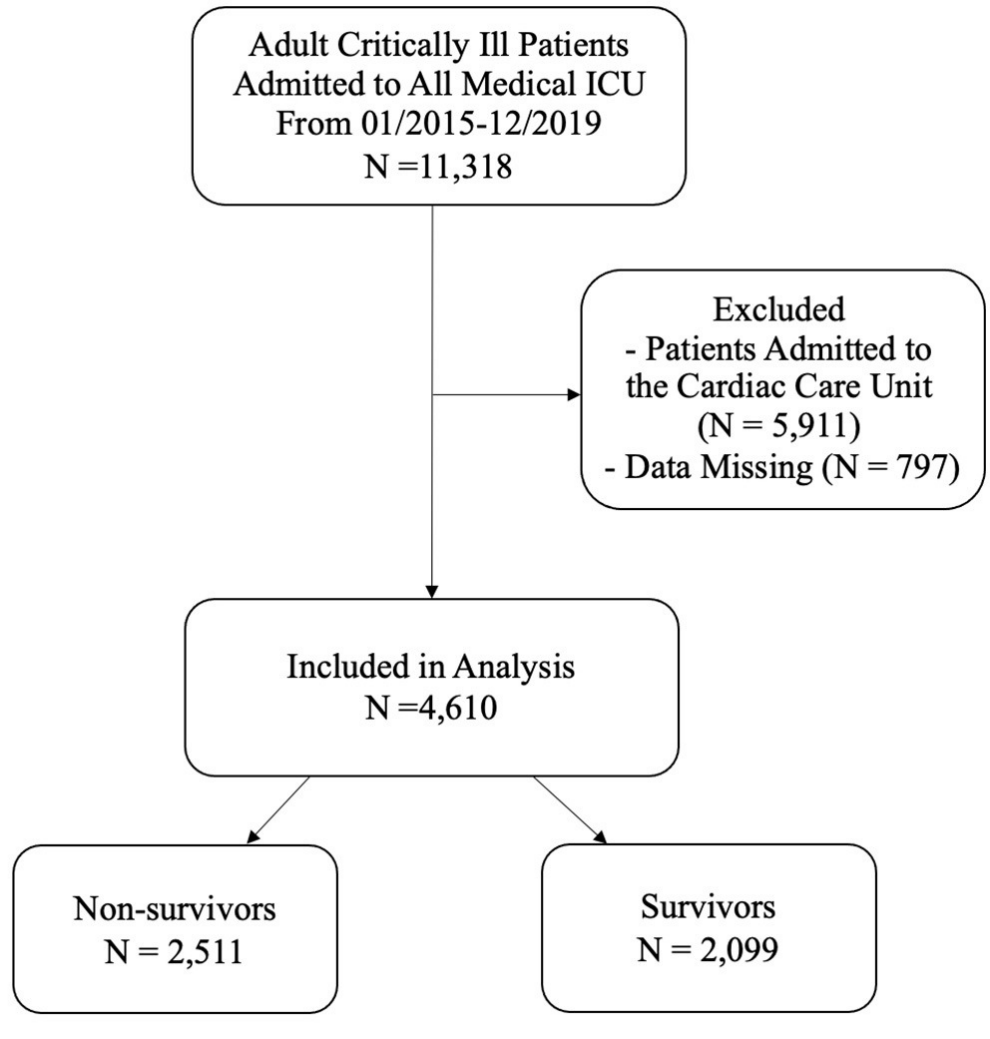
Flowchart of enrollment of subjects eligible for analyses. ICU, intensive care unit.

This retrospective cohort study was conducted at TCVGH, a tertiary care teaching hospital in central Taiwan with 1,500 beds. The TCVGH Institutional Review Board approved this study with a waiver of informed consent since this was a retrospective analysis of anonymous data (number: CE20249B). All adult patients admitted to the medical ICU from January 2015 to December 2019 were included. We used the first ICU admission as the index admission. Of the available 11,318 patients admitted to medical ICU, we excluded patients in the Cardiac Care Unit (*N* = 5,911) and patients with missing data (*N* = 797); a total of 4,610 critically ill patients were eligible for analyses (Figure 1).

### Data Source

We used two databases for this study: the clinical data warehouse at TCVGH and the cause-of-death data of the NHIRD in Taiwan. Demographic characteristics, comorbidities including Charlson Comorbidity Index (CCI), ICU admission, discharge diagnoses, daily fluid input and output, Acute Physiology and Chronic Health Evaluation (APACHE) II score, mechanical ventilation usage, renal replacement therapy (RRT) commencement, use of vasopressors, serum creatinine, and hospital length of stay were obtained from the TCVGH clinical data warehouse. The presence of shock was defined as the requirement of vasopressors for more than 1 day. Acute kidney injury (AKI) was diagnosis, and the stage was determined according to the Kidney Disease: Improving Global Outcomes (KDIGO) clinical practice guidelines for AKI (13). A patient was diagnosed with AKI if they met the criteria for AKI stage 1 or higher within the 1 week of ICU admission. The encrypted TCVGH patient identification numbers were then linked to the cause-of-death data of the NHIRD to determine their date of death up to 2019.

### Fluid Status and Study Outcome

The main exposure of interest in this study was daily fluid input, output, and balance. An intravenous or enteral fluid of any type was considered input. Output included urine, ultrafiltrate from RRT, all body fluid from drains, stool, and emesis. Fluid status was represented daily throughout the first week of ICU stay. We calculated the fluid balance for each patient as total fluid input minus total fluid output in each period. CFB of the first week was divided into two parts, the first 3 days from ICU admission and from admission day 4 to day 7 presented as CFB 1–3 and CFB 4–7, respectively. The primary outcome was the long-term all-cause mortality from the index ICU admission. The patients followed up until the ending date of NHIRD coverage or the end of the study on 31 December 2019, whichever came first. Given that National Health Insurance is a single-payer and mandatory program with a 99.9% coverage of Taiwanese population in 2019, the date of death and overall mortality in the present study should be accurate.

### Statistical Analyses

Data for categorical variables are presented as numbers and percentages; data for continuous variables are shown as means ± standard deviation. We compared the baseline characteristics of the survivors vs. non-survivors by either Student's *t*-test or the chi-squared test. Kaplan–Meier analysis was used to analyze the association between long-term mortality and the day 1–3 or 4–7 CFB status, the Kaplan–Meier curve were presented up to 1-year. A Cox proportional hazards regression model was performed to identify independent variables that predicted long-term mortality. The adjusted hazard ratio (HR) and the corresponding 95% confidence interval (CI) for each variable were presented. We used the Wald test to determine the significance of modification effect by covariates, including age, sex, shock, and presence of sepsis. Additionally, we conducted a sensitivity analysis using a subset of our cohort consisting of 3,065 patients whose AKI stage could be determined. Moreover, we investigated the interaction of shock or AKI with CFB and long-term mortality by Kaplan–Meier analysis. All reported *p*-values were two-sided and considered significant if they are <0.05. Data cleaning and analysis were performed using SAS version 9.4 (SAS Institute Inc., Cary, NC, USA).

## Results

### Baseline Characteristics of the Participants

Table 1 summarizes the baseline characteristics and clinical parameters of the participants. The mean age was 66.4 ± 16.4 years, and 2,943 patients (63.8%) were men. The reasons for ICU admission were sepsis (*n* = 2,235, 48.5%) followed by acute neurological disorder (*n* = 444, 9.6%) and respiratory disorder (*n* = 420, 9.1%). Among the total 4,610 patients, 1,291 (28%) passed away during the index admission, 1,741 (37.8%) died within 90 days, and 2,151 (46.7%) expired within 1 year after ICU admission. The patients were stratified into two groups, namely, survivors and non-survivors. The mean duration of follow-up among survivors was 2.1 ± 1.3 years. Compared with survivors, non-survivors were more likely to be older, male, with active cancer, and admitted due to sepsis or a respiratory disorder. Non-survivors compared with survivors had a significantly lower body mass index, a higher CCI, a higher APACHE II score, more mechanical ventilation, and a higher percent of shock. Besides, survivors had significantly less RRT initiated during admission compared with non-survivors. Collectively, there was high mortality among patients who survived after the index ICU admission.

**Table 1:** Patient characteristics by overall mortality (*N* = 4,610).

	**All** **(*N* = 4,610)**	**Non-survivor** **(*N* = 2,511)**	**Survivor** **(*N* = 2,099)**	***p*-value**
**Basic characteristics**				
Age, years	66.4 ± 16.4	69.7 ± 15.5	62.5 ± 16.5	<0.01
Follow-up duration, years	1.2 ± 1.3	0.4 ± 0.7	2.1 ± 1.3	<0.01
Male	2,943 (63.8%)	1,658 (66.0%)	1,285 (61.2%)	<0.01
BMI	24.4 ± 4.7	24.0 ± 4.7	24.9 ± 4.7	<0.01
Charlson comorbidity index	2.4 ± 1.6	2.7 ± 1.6	1.9 ± 1.5	<0.01
Active cancer	627 (13.6%)	523 (20.8%)	104 (5.0%)	<0.01
**Severity and managements**				
APACHE II score	25.1 ± 7.5	27.8 ± 7.1	21.9 ± 6.7	<0.01
Shock	2,134 (46.3%)	1,500 (59.7%)	634 (30.2%)	<0.01
Ventilator	3,391 (73.6%)	2,074 (82.6%)	1,317 (62.7%)	<0.01
**Renal replacement therapy (RRT)**
RRT initiated during ICU admission	738 (16%)	546 (21.7%)	192 (9.2%)	<0.01
RRT for end-stage renal disease	130 (2.8%)	69 (2.8%)	61 (2.9%)	0.75
**Reasons for ICU admission**				
Acute cardiac disorder	221 (4.8%)	42 (2.6%)	156 (7.4%)	<0.01
Acute gastrointestinal disorder	287 (6.2%)	146 (5.8%)	141 (6.7%)	0.22
Acute neurological disorder	444 (9.6%)	171 (6.8%)	273 (13.0%)	<0.01
Acute renal disorder	124 (2.7%)	57 (2.3%)	67 (3.2%)	0.06
Respiratory disorder	420 (9.1%)	289 (11.5%)	131 (6.2%)	<0.01
Sepsis	2,235 (48.5%)	1,402 (55.8%)	833 (39.7%)	<0.01
Others	879 (19.1%)	381 (15.2%)	498 (23.7%)	<0.01
**Outcomes**				
ICU-stay, days	9.9 ± 8.4	11.3 ± 8.8	8.3 ± 7.6	<0.01
Hospital-stay, days	24.2 ± 19.0	26.3 ± 19.7	21.8 ± 17.9	<0.01
Ventilator-day, days	9.7 ± 9.1	10.8 ± 9.6	8.1 ± 8.0	<0.01
In-hospital mortality	1,291 (28%)	1,291 (28%)	NA	
90-day mortality	1,741 (37.8%)	1,741 (37.8%)	NA	
1-year mortality	2,151 (46.7%)	2,151 (46.7%)	NA	

*BMI, body mass index; APACHE II, acute physiology and chronic health evaluation; RRT, renal replacement therapy; ICU, intensive care unit; NA, not applicable*.

### Daily and CFB in the First Week

Table 2 presents the breakdown of fluid balance throughout the first week of ICU stay, categorized by survival status. The fluid balance was positive on day 1 in both survivors (1473.7 ± 2985.6 ml) and non-survivors (395.7 ± 2090.1 ml). The positive to negative fluid balance transition was earlier in survivors than non-survivors, which took place on day 2 and day 3, respectively. After day 3 from admission, daily fluid balance in either group was negative. Cumulative fluid positivity was common in our cohort, with a proportion of 49.6 and 23.9% positivity in CFB 1–3 and CFB 4–7, respectively. We further evaluated the CFB 1–3 and CFB 4–7 of the survivors and non-survivors. Notably, the survivors had a negative CFB 1–3, while the non-survivors had a positive CFB 1–3. Compared with the non-survivor group, a more negative CFB 4–7 was found in the survivor group (−580 ± 2995.6 vs. −910.7 ± 2664.5, *p* < 0.01). Together, Table 2 shows that patients who survived presented a significantly lower daily fluid balance, CFB 1–3, and CFB 4–7, compared with those who died during long-term follow-up.

**Table 2:** Daily and cumulative fluid status in critically ill patients categorized by long-term mortality.

	**All (*****N*** **=** **4,610)**			**Non-survivors (*****N*** **=** **2,511)**			**Survivors (*****N*** **=** **2,099)**			***p*-value^a^**
	**Input (I)**	**Output (O)**	**IO balance**	**Input (I)**	**Output (O)**	**IO balance**	**Input (I)**	**Output (O)**	**IO balance**	
**Daily fluid status**									
Day 1	3287.6 ± 2690.9	2196.9 ± 1715.5	982.9 ± 2670.4	3745.3 ± 3056.9	2036.2 ± 1763.3	1473.7 ± 2985.6	2782.8 ± 2106.9	2371.3 ± 1644.8	395.7 ± 2090.1	<0.01
Day 2	1710.7 ± 1221.1	1841.6 ± 1376.5	−61.4 ± 1388.4	1742.3 ± 1274.2	1708.4 ± 1269.5	65.5 ± 1417.5	1674.3 ± 1156.1	1993.8 ± 1475.1	−213.2 ± 1337.3	<0.01
Day 3	1641.5 ± 970.0	2013.4 ± 1384.2	−215.7 ± 1236.4	1634.4 ± 989.7	1903.6 ± 1369.1	−136.1 ± 1301.6	1650.2 ± 945.5	2145.7 ± 1391.2	−310.9 ± 1146.6	<0.01
Day 4	1632.2 ± 1035.7	2040.5 ± 1356.7	−228.0 ± 1172.4	1619.8 ± 1048.6	1951.4 ± 1337.0	−182.3 ± 1231.7	1648.1 ± 1019	2154.3 ± 1373.5	−282.7 ± 1094.9	<0.01
Day 5	1596.0 ± 935.7	2041.3 ± 1367.4	−216.6 ± 1070.5	1595.7 ± 966.5	1957.9 ± 1351.8	−177.6 ± 1130.5	1596.4 ± 892.3	2154.5 ± 1380.8	−263.3 ± 992.2	0.01
Day 6	1619.0 ± 911.7	1979.2 ± 1285.5	−159.5 ± 985.5	1606.3 ± 942.6	1926.0 ± 1246.2	−144.2 ± 1050.0	1637.4 ± 865.3	2054.6 ± 1336.1	−177.8 ± 902.3	0.25
Day 7	1610.5 ± 954	1939.9 ± 1271.4	−126.4 ± 897.9	1624.1 ± 1014.6	1842.2 ± 1280.1	−75.9 ± 969.1	1590.1 ± 855.0	2083.5 ± 1245.3	−186.9 ± 800.4	<0.01
**Cumulative fluid status**									
Day 1–3	6070.0 ± 3971.6	5328.4 ± 3484.3	705.8 ± 3710.0	6654.6 ± 4153.4	5075.6 ± 3409.5	1403.2 ± 4096	5427.2 ± 3656.2	5603.3 ± 3544.4	−128.4 ± 2981.3	<0.01
Day 4–7	4086.0 ± 3771.3	4993.3 ± 4711.1	−730.6 ± 2854.1	4523.5 ± 3761.8	5308.0 ± 4498.8	−580 ± 2995.6	3604.4 ± 3723.7	4650.3 ± 4910.4	−910.7 ± 2664.5	<0.01

a *Comparison of IO balance between the survivors and non-survivors. Data are presented as mean ± standard deviation*.

### Both Day 1–3 and Day 4–7 Cumulative Fluid Positivity Are Correlated With Higher Long-Term Mortality

We further used CFB 1–3 and CFB 4–7 as a continuum to predict mortality. On univariate analysis, older age, male, lower BMI, CCI, APACHE II, shock, use of mechanical ventilation, active cancer, RRT initiated during ICU admission, CFB 1–3, and CFB 4–7 were significantly associated with an increased mortality risk. A multivariable model was done after adjusting for all these significant preexisting conditions and illness severity in the univariate model. Both CFB 1–3 and CFB 4–7 were independently associated with mortality (with per one liter increment, HR: 1.047 and 1.094; 95% CI 1.037–1.058 and 1.080–1.108, respectively) on multivariable analysis. Based on our results, the HR of mortality was even higher in per one liter increment of CFB 4–7 than CFB 1–3 (9.4 vs. 4.7%) in patients admitted to the medical ICU.

**Figure 2:**
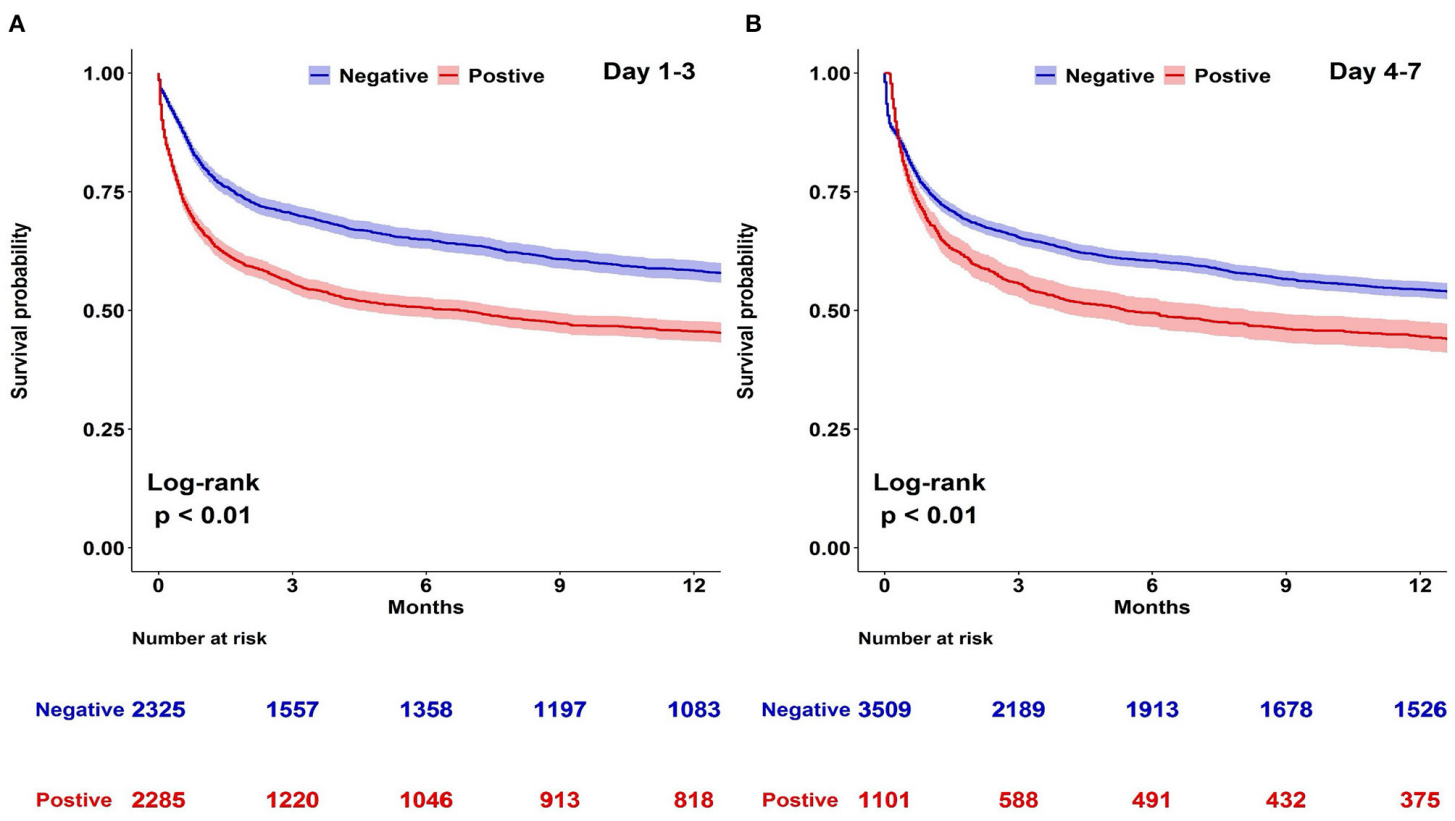
Kaplan–Meier survival curves stratified by day 1–3 or day 4–7 cumulative fluid balance (CFB). Kaplan–Meier curves for long-term survival stratified by day 1–3 or day 4–7 CFB with log-rank test model among 4,610 patients admitted to medical intensive care units. **(A)** Day 1–3 CFB: negative vs. positive. **(B)** Day 4–7 CFB: negative vs. positive.

**Figure 3:**
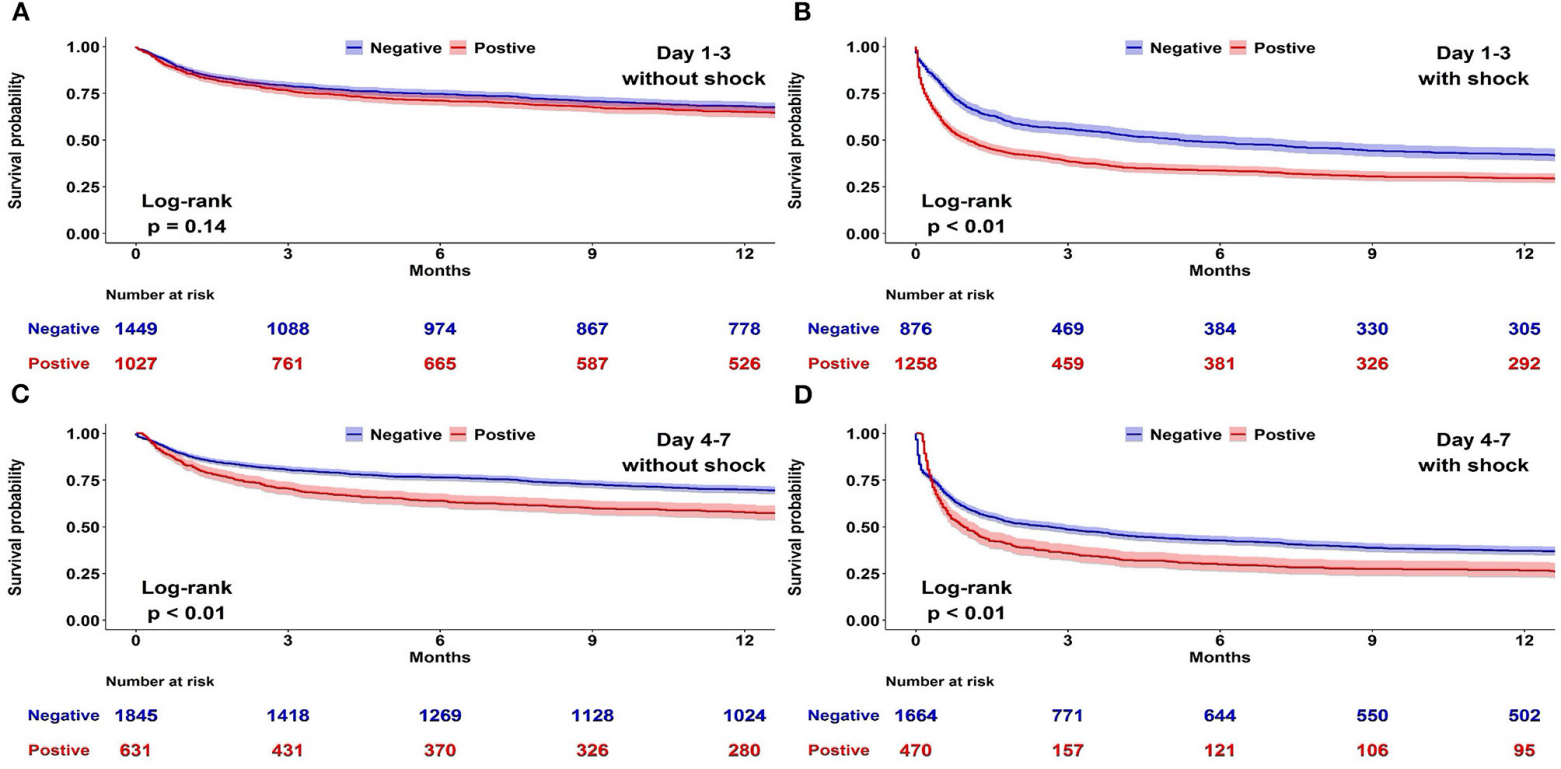
Association between positive and negative cumulative fluid balance (CFB) and long-term survival stratified by shock status. Kaplan–Meier survival curves for negative and positive CFB among 4,610 patients admitted to medical intensive care units stratified by the presence of shock. **(A)** Day 1–3 CFB in patients without shock. **(B)** Day 1–3 CFB in patients with shock. **(C)** Day 4–7 CFB in patients without shock. **(D)** Day 4–7 CFB in patients with shock.

A Kaplan–Meier analysis for 1-year survival was completed using CFB positivity or negativity as a categorical variable. We disclosed that both CFB 1–3 and CFB 4–7 positivity were associated with a higher mortality risk (Figure 2). Considering that shock status may be an important confounder of survival, we constructed Kaplan–Meier survival curves for negative and positive CFB stratified by the presence of shock (Figure 3). Shock patients with a positive CFB 1–3 were more likely to die during long-term follow-up than patients with a negative CFB 1–3. However, CFB 1–3 was not a significant predictor of mortality in non-shock patients. Compared with CFB 1–3, CFB 4–7 positivity, an indicator of post-acute stage fluid balance, correlates with higher long-term mortality in either the shock or non-shock group.

### Sensitivity Analysis

Sensitivity analysis limited the original cohort (*N* = 4,610) to a population without maintenance RRT for end-stage renal disease, stage 3 AKI at presentation in the medical ICU, or patients who stayed in the ICU <24 h, for a total analytic data set of 3,065 patients. The analysis produced robust results similar to those of the primary analysis (Supplementary Table 1 and Supplementary Figure 1 in the Supplementary Material).

### Subgroup Analysis Stratified by AKI

The population in the subset was eligible for accessing in-hospital AKI status and stages. The multivariable model in Table 3 reports that patients receiving new RRT during ICU admission were more likely to die during long-term follow-up than those without RRT (HR = 1.504; 95% CI 1.356–1.668, *p* < 0.001). Based on these results, we further explored the association of long-term outcomes and CFB in patients with different AKI status (Supplementary Figure 2 in the Supplementary Material). Likewise, patients with a positive CFB 1–3 were more likely to die than those with a negative CFB 1–3, despite their AKI status. The analysis of CFB 4–7 and long-term mortality in patients with severe AKI (KDIGO stage 2 and 3) also produced similar results. Notably, in patients without AKI or mild AKI (KDIGO stage 1), those with a positive CFB 4–7 tended to have a higher likelihood of death. However, the difference was not significant. To conclude, CFB 1–3 and CFB 4–7 may have distinct impacts on long-term survival in patients with AKI. Despite these different risks, the results point in the risk of long-term survival of severe AKI patients in favor of the negative fluid balance group.

**Table 3:** Cox proportional hazard regression analysis for long-term mortality.

**Characteristics**	**Univariable**		Multivariable^**^	
	**HR (95% CI)**	***P*-value**	**HR (95% CI)**	***P*-value**
Age, per year increment	1.017 (1.014–1.019)	<0.001	1.008 (1.006–1.011)	<0.001
Sex (male)	1.140 (1.049–1.238)	0.002	1.152 (1.060–1.252)	0.001
BMI, per 1 kg/m^2^ increment	0.976 (0.967–0.984)	<0.001	0.963 (0.955–0.972)	<0.001
CCI, per 1 score increment	1.190 (1.164–1.217)	<0.001	1.093 (1.066–1.121)	<0.001
APACHE II score, per 1 increment	1.096 (1.089–1.102)	<0.001	1.063 (1.055–1.070)	<0.001
Presence of shock	2.564 (2.367–2.778)	<0.001	1.732 (1.582–1.897)	<0.001
Use of mechanical ventilation	2.096 (1.890–2.325)	<0.001	1.199 (1.072–1.340)	0.001
Active cancer	2.907 (2.637–3.206)	<0.001	2.633 (2.380–2.912)	<0.001
RRT initiated during ICU admission	2.305 (2.095–2.536)	<0.001	1.504 (1.356–1.668)	<0.001
RRT for end-stage renal disease	0.981 (0.772–1.246)	0.875	0.938 (0.735–1.197)	0.607
CFB of day 1–3^*^	1.096 (1.085–1.106)	<0.001	1.047 (1.037–1.058)	<0.001
CFB of day 4–7^*^	1.048 (1.033–1.064)	<0.001	1.094 (1.080–1.108)	<0.001

*HR, hazard ratio; CI, confidence interval; BMI, body mass index; CCI, Charlson comorbidity index; APACHE II, acute physiology and chronic health evaluation II; RRT, renal replacement therapy; ICU, intensive care unit; CFB, cumulative fluid balance*.

* *Per 1 liter increment*.

** *Additionally adjusted for age, sex, BMI, CCI, APACHE II score, presence of shock, use of mechanical ventilation, active cancer, RRT initiated during ICU admission, CFB of day 1–3, and CFB of day 4–7*.

## Discussion

We addressed the association between early fluid balance and long-term mortality in critically ill patients and found that CFB 1–3 and CFB 4–7 positivity was independently associated with long-term mortality in patients with shock. Moreover, a positive CFB 4–7 was consistently associated with long-term mortality in critically ill patients without shock. These findings identify insights into the potential hazard of long-term mortality associated with positive fluid balance in critically ill patients and indicate future practical measures aiming a fluid status toward negative.

There is growing evidence that excess fluid administration may be detrimental to organ function (14). The FACCT trial addressed the 60-day mortality impact of liberal fluid management versus conservative therapy in patients with acute lung injury. They found no difference in mortality, but patients who received conservative fluid therapy had a shorter ventilator-day and ICU length of stay compared with those who underwent liberal fluid therapy (15). Messmer et al. performed a meta-analysis of 31 observational studies focusing on mortality association with fluid overload and found that a positive CFB 1–3 was associated with increased hospital mortality in critically ill patients (8). Several studies have further shown the clinical relevance of fluid balance in the first week among critically ill patients (5, 6, 16). Acheampong and Vincent (17) reported a positive association between the persistence of a positive fluid balance in the first week of ICU admission and high hospital mortality in 173 critically ill patients with sepsis. Similarly, Dhondup et al. (18), using a cohort comprised of 633 critically ill septic patients, reported that 61.1% of patients achieved a negative fluid balance during ICU admission, and a negative fluid balance tended to be associated with a lower 90-day mortality rate (36 vs. 44%; *p* = 0.048). In the present study, CFB 1–3 and CFB 4–7 positivity was common and highly associated with increased mortality. Collectively, this evidence indicates that a persistent positive fluid balance within the first week of ICU admission is relatively prevalent and associated with adverse outcomes among critically ill patients.

In this study, hospital mortality and 1-year mortality were 28.0% (1,291/4,610) and 46.7% (2,151/4,610), respectively. In other words, the post-acute 1-year mortality was 25.9% (860/3,319), and the prolonged mortality hazard tended to be prominent within the first 3 months. This finding was consistent with previous studies (10, 12, 19). For example, Mohr et al. recently investigated the short- and long-term outcomes of 830,721 patients with sepsis in Medicare claim database (10). They reported that hospital mortality and 90-day mortality were 20 and 48%, respectively. The long-term survival of our study coincides with prior research (Figure 2). Several studies have attempted to explore early determinants of long-term mortality in critically ill patients (12), but few studies have focused on fluid balance and long-term survival. Balakumar et al. conducted a retrospective single-center cohort study in which fluid balance during ICU stay was categorized as negative, even, or positive among critically ill patients (9). A positive fluid balance was associated with a higher 1-year mortality than an even fluid balance. Although the aforementioned finding was consistent with our data; however, the population addressed in the study conducted by Balakumar et al. was different from our cohort in the medical ICUs, such as a high proportion of surgical admissions (59.2%) rather than medical admissions, fewer patients admitted due to sepsis than our study (15 vs. 48.5%), and a lower 1-year mortality (24.4%) than in our cohort (46.7%) (9). Therefore, fluid overload tends to be a crucial issue in patients admitted to either medical or surgical ICUs.

We recognized a positive fluid balance at different stages from the first week in ICU, including CFB 1–3 and CFB 4–7, as predictors of long-term mortality (Figure 2). Differences in survival between positive and negative fluid balance were most pronounced during the first 3 months. Similarly, 3-month is currently a critical time window to define the late recovery of organ failure after critical illness, such as AKI by the Acute Disease Quality Initiative (20). We hence postulate that the recovery of organ function in survivors may potentially mitigate the initial insults related to fluid balance. Aligned with our finding, van Mourik et al. found that a positive fluid balance after reversal of septic shock was associated with high long-term mortality (21). In detail, among the 636 patients with septic shock, a higher fluid balance in the ICU stay after reversal of shock correlated with an increased 30-day and 1-year mortality. Given that most patients achieved a reversal of shock within 2–3 days, the fluid balance in the study conducted by Mourik et al. appears to be comparable with the CFB 4–7 in our study. Indeed, the post-acute fluid balance might be overlooked after recovery from critical illness. Mitchell et al. explored fluid status on ICU- discharge in 247 patients who recovered from septic shock. They found that 35% (86/247) of patients had fluid overload, defined by an increase of body weight equal or higher than 10% of body weight on ICU admission (22). They reported that patients with a fluid overload on ICU discharge were less likely to ambulate on hospital discharge and tended to be discharged to a healthcare facility instead of home (22). Taken together, patient outcomes are not only dependent on early resuscitation but also potentially affected by the fluid status after resuscitation.

Indeed, fluid management may require different approaches depending on the time course of the disease (i.e., acute vs. post-acute period). We divided early fluid balance into CFB 1–3 and CFB 4–7 to address the different roles in distinct patient groups, such as patients without shock and with shock. In shock patients, the importance of early fluid balance and the achievement of a negative fluid balance in the de-escalation phase have been extensively studied (6, 23). However, evidence gaps exist regarding CFB and long-term mortality in non-shock patients. In patients without shock, accounting for 53.7% patients in our cohort, we found that CFB 4–7, instead of CFB 1–3, was associated with long-term survival. The long-term hazard of death of positive CFB 4–7 was not affected by age, sex, shock, or presence of sepsis (Supplementary Table 2 in the Supplementary Material). Our finding highlights the importance of achieving a negative CFB in the post-acute phase even in patients without shock, which is potentially a modifiable target aimed at improved long-term outcomes.

We further point out the crucial role of fluid balance in patients with AKI. Owing to the fear that AKI might result from untreated hypovolemia, aggressive fluid administration is common in treating AKI patients. However, this practice is neither supported nor refuted by convincing clinical trials (1). The available knowledge about a higher CFB during AKI derives from observational studies focusing on short-term survival (24, 25). Thus, we specifically studied the role and the interaction of CFB with AKI and long-term survival. We found that negative CFB 1–3 had an inverse association with long-term mortality across all AKI stages (Supplementary Figure 2). In contrast, the significant survival importance of CFB 4–7 merely existed in AKI stage 2 and 3 patients, whose negative fluid balance was not easy to maintain without aggressive intervention. In a national sample of US veterans comprising 104,764 hospitalized patients (26), AKI developed in 16.3%. Most stage 1 AKI patients recovered within 2 days (71%), while slower AKI recovery was observed in patients with stage 2 and 3 AKI. The rapid restoration of renal function in less severe AKI echoes the lack of prognostic importance of CFB 4–7 in patients without AKI or mild AKI in our study. On the other hand, optimizing fluid balance in the post-acute phase among patients with severe and persistent AKI is of paramount importance and may be associated with long-term outcomes.

In the present study, the early fluid balance tended to be neutral in critically ill patients. For example, the mean CFB 1–3 was 1,403 ml and −128 ml among non-survivors and survivors, respectively. Therefore, we speculated less than half (48.5%) of enrolled patients had sepsis might account for the less requirement for aggressive fluid resuscitation. However, the enrollment of both septic and non-septic should reflect the nature of this real-world study. Moreover, the relative neutral fluid balance among patients enrolled during 2015–2019 aligns with a shift toward more restrictive fluid management in critically ill patients reported in recent trials (27).

A number of mechanistic studies have found prolonged fluid overload-associated deleterious effects, including microvascular abnormalities resulting from congestion within an encapsulated organ such as the kidney, impaired intestinal motility and nutrient absorption due to edema, dysregulated immunity possibly attributed to altered gut microbiota, and shedding of endothelial glycocalyx resulting in endothelial dysfunction (28–30). Furthermore, fluid overload may be alleviated by several measures, such as early vasopressor, higher dose of vasopressor, cautions for non-resuscitated fluid, fluid resuscitation guided by dynamic fluid responsiveness, early administration of diuretic, and protocolized diuresis (27, 31–34). Our data provides clinical evidence of a detrimental long-term impact of fluid overload and highlights that fluid balance during day 4–7 might potentially be an actionable target to alleviate fluid overload in critically ill patients.

There are limitations to be acknowledged. First, the observational design of the study does not allow the cause-and-effect relationship between CFB and outcomes to be inferred. Second, this is a single-center study and the results may not be generalizable to other populations. Nevertheless, the main cause of ICU admission and the long-term mortality rate were consistent with previous studies (11, 35). Third, the confounders, such as diuretics prescription and the dose of vasopressors, cannot be assessed in this retrospective study although we have included variables such as RRT and the presence of shock in the regression model. Besides, we defined shock as a binomial variable although the presence of shock is not an on and off phenomenon. Fourth, we could not ascertain cause-of-death and data was presented as overall mortality. Fifth, we calculated CFB during ICU stay and did not include fluid management prior to ICU admission since fluid balance charting in the ward or emergency department might not be reliable (36). Given that bias could have resulted in underestimating the value of fluid overload, such a misclassification is more likely to influence the results toward the null hypothesis. Additionally, the individual physician made the decision of fluid therapy that could lead to a confounding effect. However, with dedicated intensivists and fluid management in accordance with the guidelines, it might partly mitigate the concern.

## Conclusions

We linked two databases to address the long-term mortality association of fluid balance with the first week in critically ill patients. We found that patients with a positive CFB were independently associated with higher long-term mortality than those with a negative CFB. Patients with per one-liter increment of CFB 1–3 and CFB 4–7 significantly bore 4.7 and 9.4% risk odds of long-term mortality. Notably, we identified the role of a positive CFB 4–7 on long-term mortality in patients without shock or in those with severe AKI, indicating that CFB 4–7 might be a potentially modifiable factor to improve long-term outcomes. Further prospective studies focusing on both early fluid balance and post-acute fluid balance are warranted to validate our findings.

## Data Availability Statement

The raw data supporting the conclusions of this article will be made available by the authors, without undue reservation.

## Ethics Statement

The studies involving human participants were reviewed and approved by the Institutional Review Board of Taichung Veterans General Hospital (TCVGH: SE20249B). The Ethics Committee waived the requirement of written informed consent for participation.

## Author Contributions

T-JW, C-HC, C-LW, and W-CC: study concept and design. C-LW: study coordination. T-JW, C-LW, K-CP, L-TW, M-SW, and W-CC: acquisition of data. K-CP, L-TW, C-ML, and W-CC: statistical analysis. T-JW, C-TH, C-HC, C-LW, and W-CC: interpretation of data. T-JW and W-CC: drafting the manuscript. All authors read and approved the final manuscript.

## Funding

This study was supported by Ministry of Science and Technology Taiwan (MOST 109-2321-B-075A-001), Taichung Veterans General Hospital (TCVGH-1114402B) and Veterans General Hospitals and the University System of Taiwan Joint Research Program (VGHUST110-G2-1-2). The funders had no role in the study design, data collection and analysis, decision to publish, or preparation of the manuscript.

## Conflict of Interest

The authors declare that the research was conducted in the absence of any commercial or financial relationships that could be construed as a potential conflict of interest.

## Publisher's Note

All claims expressed in this article are solely those of the authors and do not necessarily represent those of their affiliated organizations, or those of the publisher, the editors and the reviewers. Any product that may be evaluated in this article, or claim that may be made by its manufacturer, is not guaranteed or endorsed by the publisher.

## Abbreviations

AKI: Acute kidney injury
APACHE: Acute Physiology and Chronic Health Evaluation
CCI: Charlson Comorbidity Index
CFB: Cumulative fluid balance
CFB 1-3: Cumulative fluid balance Day 1-3
CFB 4-7: Cumulative fluid balance Day 4-7
CI: Confident interval
HR: Hazard ratio
ICU: Intensive care unit
KDIGO: Kidney Disease: Improving Global Outcomes
NHIRD: National Health Insurance Research Database
RRT: Renal replacement therapy
TCVGH: Taichung Veterans General Hospital.
