# Supplementary material for: A Positive Fluid Balance in the First Week Was Associated With Increased Long-Term Mortality in Critically Ill Patients: A Retrospective Cohort Study
Source: Front Med (Lausanne). 2022 Mar 3;9:727103. doi: 10.3389/fmed.2022.727103 (PMC8927621; doi:10.3389/fmed.2022.727103)

Supplemental data

Supplemental table 1. Patient characteristics of the 3,065 patients enrolled for acute kidney injury and long-term mortality analyses

	All (N = 3,065)	Non-survivor (N = 1,596)	Survivor (N = 1,469)	*p* value
**Basic characteristics**				
Age, years	67.0±16.3	70.3±15.4	63.6±16.6	<0.01
Survival years	1.13±1.2	0.4±0.7	1.9±1.2	<0.01
Male	1969 (64.2%)	1080 (67.7%)	889 (60.5%)	<0.01
BMI	24.2±4.7	23.6±4.7	24.7±4.8	<0.01
Charlson Comorbidity Index	2.3±1.6	2.7±1.6	2.0±1.5	<0.01
Active cancer	449 (14.6%)	369 (23.1%)	80 (5.5%)	<0.01
**Severity and managements**				
APACHE II score	24.3±7.1	26.7±6.7	21.7±6.6	<0.01
Shock	1359 (44.3%)	880 (55.1%)	479 (32.6%)	<0.01
Ventilator	2483 (81.0%)	1421 (89.0%)	1062 (72.3%)	<0.01
**Reasons for ICU admission**				
Acute cardiac disorder	97 (3.2%)	31 (1.9%)	66 (4.5%)	<0.01
Acute gastrointestinal disorder	196 (6.4%)	92 (5.8%)	104 (7.1%)	0.14
Acute neurological disorder	364 (11.9%)	129 (8.1%)	235 (16.0%)	<0.01
Acute renal disorder	43 (1.4%)	18 (1.1%)	25 (1.7%)	0.18
Respiratory disorder	295 (9.6%)	195 (12.2%)	100 (6.8%)	<0.01
Sepsis	1614 (52.7%)	947 (59.3%)	667 (45.4%)	<0.01
Others	456 (14.9%)	184 (11.5%)	272 (18.5%)	<0.01
**AKI status***				
No AKI	490 (16.0%)	135 (8.5%)	355 (24.2%)	<0.01
Stage 1	890 (29.0%)	503 (31.5%)	387 (26.3%)	<0.01
Stage 2	1283 (41.9%)	698 (43.7%)	585 (39.8%)	0.03
Stage 3	402 (13.1%)	260 (16.3%)	142 (9.7%)	<0.01
**Outcomes**				
ICU-stay, days	10.8±8.2	12.4±8.5	9.1±7.5	<0.01
Hospital-stay, days	26.0±18.5	28.6±19.2	23.2±17.3	<0.01
Ventilator-day, days	10.1±9.1	11.6±9.5	8.1±8.1	<0.01
In-hospital mortality	736 (24.0%)	736 (24.0%)	NA	
90-day mortality	1078 (35.2%)	1078 (35.2%)	NA	
1-year mortality	1363 (44.5%)	1363 (44.5%)	NA	

Abbreviations: BMI, body mass index; APACHE II, acute physiology and chronic health evaluation II; ICU, intensive

care unit; AKI, acute kidney injury; NA, not applicable.

*AKI diagnosis was made and stages were assigned based on the current guidelines published by the Kidney Disease Improving Global Outcomes (KDIGO) group

Supplemental table 2. Modification effect of variables on the association between early fluid balance and risk of long-term mortality

Variables	Day 1-3 Fluid balance		Day 4-7 Fluid balance	
	**Adjusted HR (95% CI)**	***P* value**	**Adjusted HR (95% CI)**	***P* value**
**Age group**		0.272		0.446
≤ 50 years	1.573 (1.252-1.977)		1.308 (1.011-1.691)	
> 50 years	1.453 (1.336-1.580)		1.254 (1.141-1.379)	
**Sex**		0.744		0.300
Female	1.440 (1.258-1.648)		1.347 (1.161-1.562)	
Male	1.493 (1.355-1.645)		1.232 (1.102-1.376)	
**Presence of shock**		<0.001		0.997
No	1.098 (0.969-1.244)		1.393 (1.217-1.594)	
Yes	1.504 (1.354-1.670)		1.297 (1.152-1.460)	
**Presence of sepsis**		0.012		0.183
No	1.317 (1.147-1.512)		1.370 (1.175-1.599)	
Yes	1.640 (1.490-1.805)		1.212 (1.087-1.351)	

Abbreviations: HR, hazard ratio; CI, confidence interval.

Figure S1. Survival by day 1–3 or day 4–7 cumulative fluid balance (CFB).

Kaplan–Meier curves for long-term survival stratified by day 1–3 or day 4–7 CFB with log-rank test model among 3,065 patients admitted to medical intensive care units. (A) Day 1–3 CFB: negative vs. positive. (B) Day 4–7 CFB: negative vs. positive.

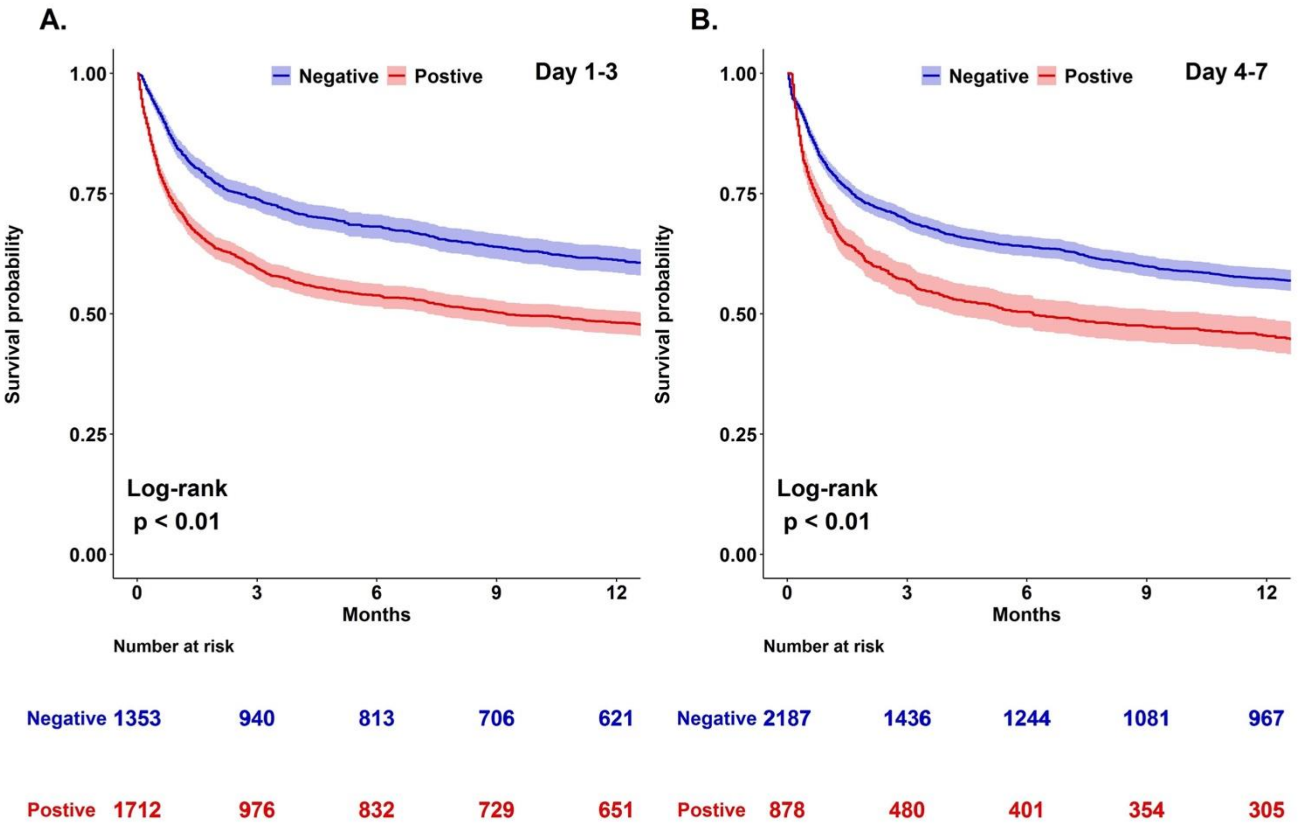

Figure S2. Survival by cumulative fluid balance (CFB) and acute kidney injury (AKI) stage. Kaplan–Meier survival curves for negative (blue) and positive (red) CFB among 3,065 patients admitted to medical intensive care units stratified by status of AKI. (A) Day 1–3 CFB in patients without AKI. (B) Day 1–3 CFB in patients with AKI stage 1. (C) Day 1–3 CFB in patients with AKI stage 2 and 3. (D) Day 4–7 CFB in patients without AKI. (E) Day 4–7 CFB in patients with AKI stage 1. (D) Day 4–7 CFB in patients with AKI stage 2 and 3.

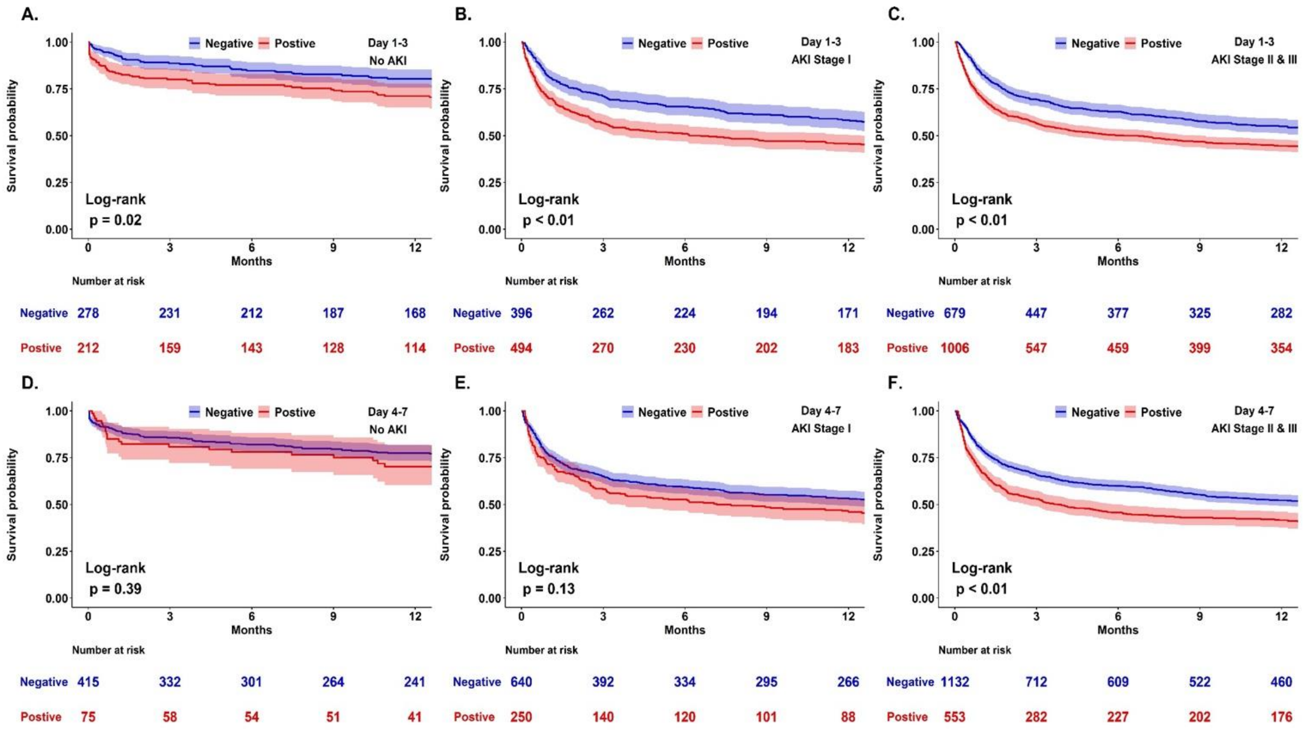

Supplement: Supplementary file 1 [file Data_Sheet_1.pdf]
